# Current Approaches for Image Fusion of Histological Data with Computed Tomography and Magnetic Resonance Imaging

**DOI:** 10.1155/2022/6765895

**Published:** 2022-11-09

**Authors:** Philipp Nolte, Christian Dullin, Angelika Svetlove, Marcel Brettmacher, Christoph Rußmann, Arndt F. Schilling, Frauke Alves, Bernd Stock

**Affiliations:** ^1^Faculty of Engineering and Health, University of Applied Sciences and Arts, Goettingen 37085, Germany; ^2^Institute for Diagnostic and Interventional Radiology, University Medical Center Goettingen, Goettingen 37075, Germany; ^3^Department of Trauma Surgery, Orthopedics and Plastic Surgery, University Medical Center Goettingen, Gottingen 37075, Germany; ^4^Translational Molecular Imaging, Max-Planck Institute for Multidisciplinary Sciences, City Campus, 37075 Goettingen, Germany; ^5^Department for Diagnostic and Interventional Radiology, University Hospital Heidelberg, Heidelberg 69120, Germany; ^6^Brigham and Women's Hospital, Harvard Medical School, Boston 02155, MA, USA

## Abstract

Classical analysis of biological samples requires the destruction of the tissue's integrity by cutting or grinding it down to thin slices for (Immuno)-histochemical staining and microscopic analysis. Despite high specificity, encoded in the stained 2D section of the whole tissue, the structural information, especially 3D information, is limited. Computed tomography (CT) or magnetic resonance imaging (MRI) scans performed prior to sectioning in combination with image registration algorithms provide an opportunity to regain access to morphological characteristics as well as to relate histological findings to the 3D structure of the local tissue environment. This review provides a summary of prevalent literature addressing the problem of multimodal coregistration of hard- and soft-tissue in microscopy and tomography. Grouped according to the complexity of the dimensions, including image-to-volume (2D ⟶ 3D), image-to-image (2D ⟶ 2D), and volume-to-volume (3D ⟶ 3D), selected currently applied approaches are investigated by comparing the method accuracy with respect to the limiting resolution of the tomography. Correlation of multimodal imaging could position itself as a useful tool allowing for precise histological diagnostic and allow the a priori planning of tissue extraction like biopsies.

## 1. Introduction

Examination of pathological alterations in human tissue by histology is an integral part of clinical routine. Countless staining protocols and immunohistochemistry applications have been developed for histology, thus enabling the identification of specific cell types, subcellular structures, substrates, and disease biomarkers, which renders this approach extremely versatile.

However, histological evaluation requires access to tissue specimens. Such specimens are usually obtained by biopsy, with the associated discomfort and risks. Based on the specimen type, histology can be divided into (i) soft-tissue histology in which the specimen is typically embedded in paraffin and cut with a microtome utilizing a static blade and (ii) hard-tissue histology for which the samples are embedded in resin, cut with a diamond saw, and then grinded down to a slice thin enough to be stained and imaged under a microscope. In both scenarios, the cutting is typically done lacking any a priori knowledge regarding the position of the region of interest (ROI), which in turn limits the prognostic value of the histology analysis and/or leads to a very time-consuming serial cutting of the specimen. In hard-tissue histology, this situation is further complicated by the fact that the specimens are typically opaque and cut-grind techniques result in the loss of a substantial percentage of the material. Since classical histology is based on the evaluation of micrometer thin tissue slices by microscopy, the biopsies are typically only sparsely sampled, bearing the risk of missing important key aspects. In addition, intrinsically three-dimensional features such as metastatic volumes, fiber orientations, and so on cannot be effectively accessed in planar slices. This can, in theory, be solved with serial sectioning and 3D reconstruction, but this is extremely labor-intensive.

A solution to some of these current shortcomings could be a combination of histology with high-resolution 3D-imaging techniques such as micro-CT and micro-MRI performed prior to sectioning. This would allow histology to be supplemented with measures of 3D features and to spatially localize the findings of histology within the local 3D tissue environment thereby raising the prognostic value of the analysis. Furthermore, this combination would enable “guided sectioning” as demonstrated by Albers et al. [1] by using micro-CT scans of lung tissue to plan the subsequent sectioning and isolation of the ROI. Rau et al. [2] embedded artificial markers together with their tissue specimen in order to accurately reconstruct the extracted human temporal bone based on landmark-registration. They argue that the presence of fiducial markers and an additional planning phase before cutting can beneficially aid image-guided sectioning and computer-aided surgery.

**Figure 1:**
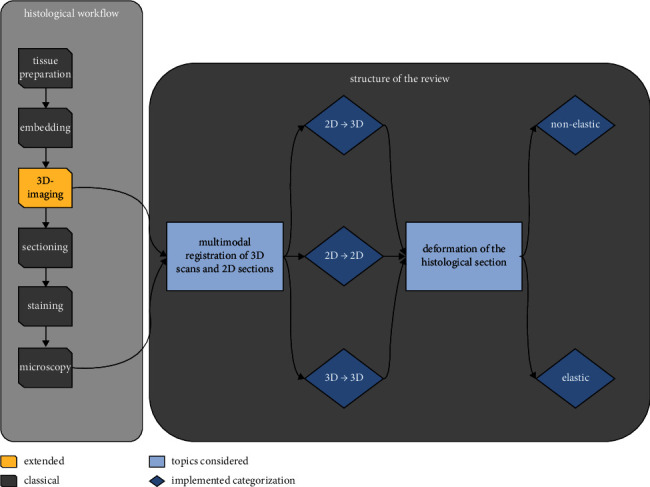
An overview of the presented review concerning multimodal registration of 3D scans and 2D sections. The methods described here extend the classical histological workflow with 3D imaging before sectioning. Thus, image representations of the tissue are present in two modalities. The research considered is grouped according to the dimensionality of the addressed problem into slice-to-volume (2D ⟶ 3D), slice-to-slice (2D ⟶ 2D), and volume-to-volume (3D ⟶ 3D) registration. Each category is further subdivided into elastic and non-elastic algorithms. This results in a specialized selection of algorithms that match and remedy the corresponding severity of deformations introduced during the sectioning process.

One of the main problems of image fusion between histology and 3D-imaging is that the process of sectioning (especially in soft-tissue) can introduce nonuniform deformation which needs to be compensated by the employed registration pipeline. To account for this problem, a vast variety of combinations of different imaging techniques and registration strategies have been proposed. In the scope of this review, we assessed and grouped these strategies according to their dimensionality in three different chapters: (i) extracting a corresponding 2D cut section from the 3D data set for subsequent 2D-2D registration with the histological slice, (ii) placing the 2D histological slice into a 3D data set, and (iii) fusion of 3D serial sectioning with the 3D-imaging data. There is also a large variety of algorithms that are used in each of the different scenarios, starting from a simple manual registration of the data as performed by Mourad et al. [3] for the fusion of histology and micro-CT down to elastic image registration as employed by, for instance, Albers et al. [1, 4, 5]. Thus, the three chapters are subdivided into applications of elastic and nonelastic methods.  Figure 1 illustrates the typical pipeline of combined 3D-imaging and histological tissue analysis as well as the discussed structure of the review.

The reported relative registration accuracies are compared based on the resolution of the data to facilitate finding a suitable strategy for the task at hand.

**Figure 2:**
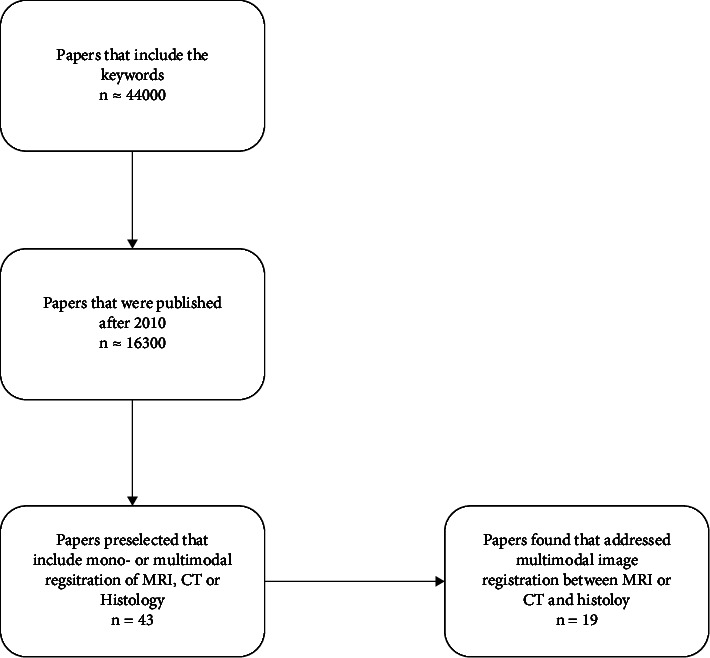
Visualization of the process behind the inclusion of the selected literature. Starting with over 44000 initial hits for the stated search terms, 16300 were published after 2010 and considered for further investigation. From this group, we identified 43 papers concerning image registration in the context of CT, MRI, and histology. From this group, we further extracted 19 that addressed multimodal registration.

We started our search for literature intending to find approaches concerning the terms “CT,” “MRI,” and “histology” in synergy with the topic of “multimodal registration.” In order to keep the number of publications tractable, we excluded publications printed before 2010. The search was conducted through Google Scholar and Web of Science. This resulted in a preselection of forty-three papers, which we investigated for solutions to the problem of registration of hard- or soft-tissue with CT or MRI scans. Out of these forty-three papers, we identified 19 that addressed the problem of multimodal image registration between MRI or CT and histology and thus met our criteria and were considered for this review. Research addressing monomodal registration was not excluded from our scope. The described process is visualized in the literature including flowchart depicted in Figure 2.

## 2. Multimodal Image Registration

**Figure 3:**
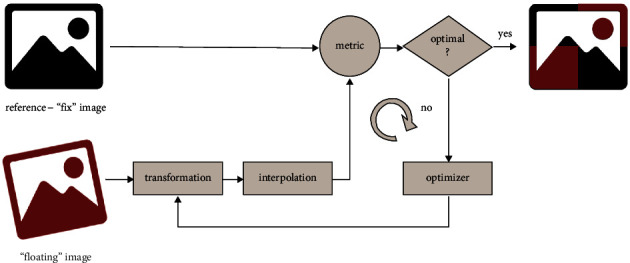
A flow diagram of a basic registration approach. The reference or fixed image is used as a ground truth, while the floating image is iteratively altered by transformation and alteration until the optimal overlay is achieved. The registration process is completed when the optimal value for the similarity (based on a specific metric) between the two images is found.

The typical procedure of image registration is illustrated in Figure 3: in our case, one data set (in our case, the 3D data of CT or MRI or a virtual slice of those) is considered to be the ground truth and is not modified during the entire process, usually referred to as “fixed image” [6–8]. The second dataset (here the histology slice or a set of histology slices) is deformed during the process to “optimally” match the underlying fixed image; this image is usually called the “floating image.” Registration, as described here, has been implemented in prominent software libraries dedicated to image processing like elastic [9]. The presented algorithms differ mainly in two key aspects: (i) which type of deformation they allow for the floating image and (ii) which criteria (metric) they use to assess matching quality.

An important aspect of image registration is how the two images are compared once an ideal transformation of the floating image has been achieved. Two main strategies are commonly employed: (a) using the entire image information by, for instance, cross-correlation or mutual information and (b) using landmarks (either intrinsic ones if, for instance, implants are present in the data or generated by algorithms such as speeded up robust features-SURF or scale-invariant feature transform-SIF, to only name a few). In the first case, the registration process is typically robust and tolerates partially missing data in the images or the presence of artifacts. However, both data sets have to have a large degree of similarity, like, for instance, CT and chemically stained histology. The second case can also deal with vastly different image content like CT and immunohistochemistry, but identification of good landmarks may be challenging.

One drawback to comparing the performance of different registration algorithms or picking the optimal procedure for a given task is the lack of standardized quality measures. Typically, the same metric used for the registration process is used to perform quality assurance. Especially in the case of landmark-based approaches, an ideal match of the landmarks does not necessarily imply optimal registration of the entire data. A large variety of measures can be utilized to assess the quality of a given registration approach, with the simplest being a sheer calculation of the translational and rotational errors in plain units of distance and tilt. Other approaches estimate the L1-distance between two points in the target and moving images, as implemented by [10, 11]. If of interest, statistical metrics associated with the used imaging technique like the Dice index [12] (also called F1-Score) are used.

In order to provide some means of comparison of the presented algorithms, we calculated relative accuracies based on the spatial resolution of the applied imaging techniques (although not all publications listed those parameters). In a later section, we compiled these values into three tables according to the dimension of the registration approach.

In the following, we will discuss registration strategies loosely grouped according to the dimension of the input data.

### 2.1. Slice-to-Volume Registration (2D ⟶ 3D)

**Figure 4:**
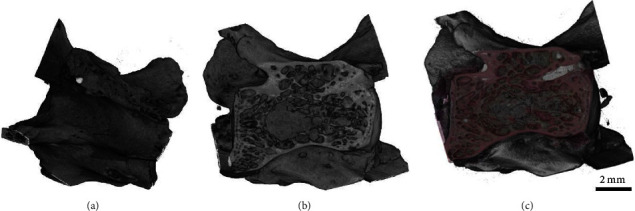
Exemplary fusion of a CT scan with a histological slide. Representative images of a rat vertebrae by CT. (a) The plane of interest in the scan. (b) The fusion of the scans allows for the combination of specific histological features present in the stained tissue of the 2D section and (c) the global geometrical structure of the specimen found in the CT scan. The correlation was realized by superimposing both the estimated CT-plane (b) and the corresponding histological section (c).

This approach may seem most straightforward in terms of combining histology with CT or MRI. The aim is to place the histology section into the 3D context of CT or MRI as depicted in Figure 4 for an application combining a Sanderson's Rapid Bone and Van Gieson-stained section with a micro-CT scan of a resin embedded vertebrae of a rat.  Figure 4(a) illustrates the whole scan, which was iterated through in search of the best fitting cutting plane (Figure 4(b)). Finally, both the histological section and the cut CT volume are fused in Figure 4(c).

Following the CT scan, the embedded vertebrae were sectioned using a combination of a diamond-cut grinder and a LASER microtome. The resulting section was stained with Sanderson's Rapid Bone and Van Gieson, scanned with a microscope (Axiovert 200 inverted microscope, Zeiss) and then manually positioned in the 3D data set visualized in VGStudioMax (Volume Graphics), a 3D rendering and analysis software. Figure 4(c) indicates a nearly precise match which proves two aspects: firstly the cutting plane reflects a “strict” plane in the 3D data set and, therefore, allows to reduce the problem to a 2D-2D registration problem, if the correct virtual plane in the 3D data set can be identified and secondly that hard-tissue embedded in resin is not subjected to relevant nonuniform deformations during the cutting process which eases the registration process as only a ridged body transformation needs to be found. In terms of paraffin-embedded, soft-tissue sectioning results in local deformations especially in porous tissue like the lung as reported by Albers et al. [1]. However, even in lung tissue, the deformation is mainly restricted to the cutting plane due to the nature of the cutting system. Thus, in all cases, the approach can be split into the identification of the in silico cutting plane in the 3D data set and subsequent registration with the histological slice. Albers et al. used elastic image registration, treating the a priori acquired micro-CT as ground truth for that [5]. In order to reduce deformations between the individual sectioned planes, the integration of 3D printing slicers or cutting boxes has been proposed [13], where the sectioning process is optimized through the inclusion of a 3D segmented model of the tissue. This model is then used as a reference for the creation of a specimen cutting in a 3D modelling or computer-aided design program and subsequent printing.

Depending on how strongly the histological image is distorted in comparison to the in-silico plane, 2D-registration algorithms with different degrees of freedom need to be applied. This can be loosely grouped into nonelastic, i.e., rigid or affine, and elastic registration algorithms as pointed out by [6] and confirmed to be still valid later by the same authors in [7]. Thus, the main problem is identifying the in-silico plane. For this purpose, multiple strategies are proposed, ranging from manual identification of the plane by Albers et al. [1] to complete automatic detection. In many cases, deformation or loss of tissue in the histological slide complicates the search for the corresponding plane in the 3D volume. Due to possible shifts in the slicing of the tissue, the problem cannot therefore be simplified to a 2D ⟶ 2D transformation. These shifts are commonly prevalent in soft-tissue sections as a result of the physical cut of the microtome. Hence, a two-step approach with a preliminary coarse and subsequent fine alignment was proposed [11, 14, 15]. The plane in the 3D volume is coarsely aligned first by matching to a group of candidate slices, followed by refined correction of plane shifts and tilts. The initial alignment of the two modalities may be performed feature-free by the alignment of corresponding extrinsic markers and matched pixel/voxel intensities.

#### 2.1.1. Nonelastic Approaches for Slice-to-Volume Registration (2D ⟶ 3D)

Lundin et al. [14] sampled a group of candidate planes from micro-CT scans of porcine vertebrae trabecular bones with the corresponding specific orientation parameters. Candidate planes were determined by searching for the maximum number of identified Harris corner detector key points detected in the histological image. From each key point, a descriptor vector is generated with a simplified version of the histogram of oriented gradients (HoG) algorithm [16] that is matched with a given CT-plane key point through an implementation of the nearest-neighbor algorithms. The binarized histological slice was then rigidly aligned by its center at a low resolution with all candidate planes based on the matched key points and optimized through RANSAC. The rotation was estimated based on pixel-intensity values using the Radon transform [17, 18]. The Radon transform is based on measuring the length of lines between two points and returns the perpendicular distance between the origin and the destination as well as the angle between the line and the *y*-axis. The latter was used to estimate the rotation. Through calculating the sum of the edge distances [17] between a CT-plane and the histological slice in an inverted order, a cost function was established in order to optimize the Radon transform.

If extrinsic markers like implants are present, segmentation-based approaches to estimate coarse positioning can be considered [11, 15]. Based and expanding on the work by Sarve et al. [15], Becker et al. [11] used Chamfer matching [17] to preliminarily align images of specimens containing dental implants on the basis of thresholding. Due to the inflexible nature of the implant, initialization was approached through alignment of the corresponding axis, which was approximated through principal component analysis (PCA) [19]. The edge vectors of the histology and *µ*CT images were centered and saved in a matrix. From this matrix, the covariance matrix and eigenvalue composition were calculated, where the eigenvector with the largest eigenvalue yielded an approximation of the implant axis. Using the implant axis as an initialization, an extraction of adjacent slices was conducted, following an extraction of adjacent slices. Candidate slices were then determined by the estimation of the optimal Chamfer distance [17], a measure to identify the nearest edges between two planes, and the smallest root-mean-squared error (RMSE) was approximated. In order to further refine the initial alignment, candidate slices were identified through a rotation in 5-degree steps orthogonal to the identified implant axis. A subsequent 10-degree rotation in 1-degree steps was performed at the positions that resulted in the highest similarity. The similarity of adjacent slices was quantified using an alignment score (*L*-score) that was composed of the averaged L1-Norm between two aligned pixels. Plane parameters were extracted based on the optimal *L*-score, which was considered to be equal to a coarse alignment of the histological image in the CT volume.

**Figure 5:**
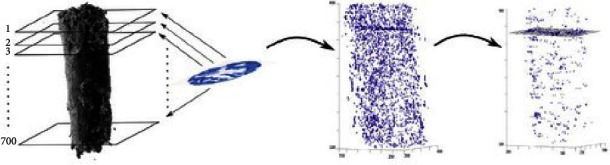
Exemplary workflow of a feature-based approach by Chicherova et al. [20] based on data originating from extracted human jawbones [29]. First, SURF-feature points (blue dots) were detected in all 700 planes of the CT scan and the histological image (left). These descriptors were then compared in a higher-dimensional space (middle). Matching descriptors of both modalities form a plane that was used as a basis to estimate the position of the histological section in the CT volume (right). The plane was then further optimized by utilizing RANSAC (right). Reprinted by permission from Springer Nature customer service center GmbH: patch-based techniques in medical imaging. Patch-MI 2017. Lecture notes in Computer Science, vol 10530, Histology to *μ*CT data matching using landmarks and a density-biased RANSAC, Chicherova, N., Fundana, K., Müller, B., Cattin, P.C., Copyright© 2014 springer international publishing Switzerland.

In the case of registration methods based on extracted features in both modalities, a prime alignment step can be neglected in favor of grouping mechanisms performed in a higher dimensionality space [14, 20–22]. Feature points can be extracted from each image and represented as vector points. Based on the proximity of these points in the vector space, an affiliation and subsequent geometric dependency can be detected. These descriptors can, for example, be obtained by utilizing a Harris corner detector [23] and HoG [16] as presented by [14] or SURF [24] and SIFT [25] algorithms shown by [20], who neglected the initial alignment. Corresponding feature clouds were matched by either calculating the Euclidean distance or by utilizing a variant of the nearest-neighbor algorithm [25, 26], where data points are grouped together based on their proximity to one another. The validity of these detected matches was verified by a variant of the random sample consensus (RANSAC) optimization scheme [27, 28], which resulted in transformations with six degrees of freedom. RANSAC arbitrarily defines a minimum number of data points sufficient to describe the target shape, with three points as the minimum representation of a plane. In an iterative process, the number of points inside a given distance interval, so-called inliners, is counted. The parameters of the plane are then updated until the number of inliers decreases. If data points that do not fit the plane, called outliers, are still present at that point, the process is started again with a different subset of points. An example of a feature-based approach is depicted in Figure 5.

Given that both images have been preliminarily matched regarding translation and rotation, fine alignment can be achieved through intensity-threshold-based approaches like simulated annealing [30] for fine affine alignment of the histology image onto a predetermined slice in the volume as presented by [11]. To refine the coarse alignment estimated by the chamfer distance, they moved on to find the transformation parameters that yielded the optimal alignment of both modalities. Taking inspiration from annealing in metallurgy, a global optimal position was searched in a slow iterative process instead of a fast estimation of a local minimum [30–33]. This was achieved by predicting if a higher alignment score, calculated through an alteration of the L1-norm between two pixels (*L* score, see description above), can be achieved between the fixed histological slide and two adjacent CT-planes predicted by the initial placement. Simulated annealing yields six degrees of freedom in translation and rotation, allowing for transformation with the goal of maximizing the *L*-score and thus minimizing the offset between two given sections of both modalities. While this approach produces acceptable results (median L score: 91 out of 100, CT isotropic nominal resolution: 8.6 *µ*m), they argue that their results can be hardly improved due to limiting segmentation from artifacts introduced during preprocessing of the tissue or irregularity of the staining. This workflow by Becker et al. [11] is highly dependent on the presence of distinguishable artifacts like implants and can be categorized as a landmark-based registration approach. Image segmentation and the manual determination of a suitable threshold are the method's bottlenecks. The applicability of this method to other problems is therefore not fully guaranteed.

Utilizing the initial placement of the histological slide in the volume, Lundin et al. [14] continuously affinely registered several adjacent parallel planes with the image, relying on feature points detected by a Harris corner detector [23]. Each key point was subsequently utilized to extract descriptor vectors, which were then matched by employing the nearest-neighbor matching algorithm. Depending on whether the validity of the match was determined by the means of optimal RANSAC [28], an affine registration was estimated according to the greatest number of valid matches. The authors state that the presented approach is different from previously conducted research due to the fully automated plane estimation and its applicability to distinguish highly structured objects. Employing simulated and real 2D data, an average orientation error of 0.6° was found. The target registration error, i.e., the distance between two manually annotated points in target and moving images, was computed based on manually defined landmarks and determined to be 106.3 *µ*m (corresponding to about 10 pixels in the difference between the target and moving landmark). The validation was performed without any prior knowledge of the geometrical shape of the volume. Two different varieties of CT scans were used: one with a lower resolution of 85.6 *µ*m for coarse alignment and one with a resolution of 21.4 *µ*m for finer alignment (nominal CT resolution: 10.7 *µ*m), while the pixel resolution of the histological image was measured to be 2.55 *µ*m. A clear reduced performance was observed for nonartificial data due to deformations introduced to the specimen during preprocessing, which could be accounted for by introducing countermeasures for local deformations.

As described before, thresholding as a preprocessing step for alignment may not apply to all datasets or may even possibly limit the overall superimposing performance [11]. Chicherova et al. [20] proposed a feature-based approach to automatically and without any a priori knowledge rigidly align a histological slide to an arbitrary plane in the CT volume of jawbones based on their earlier work [29]. Employing a SURF detector [24], a subset of feature points and respective descriptor vectors were determined from both histological images and predetermined slices of the micro-CT volume. Corresponding feature points were then chosen by calculating the Euclidean distance through a second nearest-neighbor criteria and a given threshold to validate a candidate match. This process was then repeated for all slices of the micro-CT dataset. To correctly define the fitting plane, a RANSAC optimization scheme [27] was used to obtain a descriptive four-dimensional normal vector. Chicherova et al. [20] found an average error distance of 0.25 mm for correctly matched slices, leaving room for improvement with a 75% success rate. They analyzed specimens smaller than a tube of 3 mm in diameter and 12 mm in length, which were scanned with a resolution of about 4 *µ*m (depending on the specimen) [29]. The authors state that they plan to develop a more suitable feature detector with an elastic matching approach. In subsequent research, the described workflow was adapted by Khimchenko et al. [21] with the additional use of the Demon registration tool [34]. By affinely deforming the tomographic image, they aligned both modalities and verified their results through comparison to expert 2D ⟶ 3D registration. They show that they improved their former workflow for their specific test cases, yielding radial and longitudinal stretches of 6% and 15%, respectively. Overall, they state that the resolution of the micro-CT was limited to the size of the focal spot (around 0.9 *µ*m) thus limiting the overall achievable registration results. Through the comparison of their results with the expert-based ground truth, a mean difference of 4 *µ*m between the characteristic landmarks and the automatic and manual registration planes was observed. Chicherova et al. improved their work in a later publication [22] through the addition of an elastic registration step and normalized mutual information. Their work will be presented in the following chapter.

#### 2.1.2. Elastic Approaches for Slice-to-Volume Registration (2D ⟶ 3D)

With an elastic optimization scheme based on normalized mutual information, Chicherova et al. extended their previous workflow in [22]. Instead of solving the problem of finding an optimal elastic deformation through B-Splines, they opted for a Legendre polynomial, which considered the entire space of possible deformations instead of a piecewise deformation model. In an iterative step, the plane obtained by the RANSAC scheme is subsequently altered with the goal of optimizing the alignment. The coefficients are calculated as the result of the least square solution of a system of linear equations. Through an optimization framework, the coefficients are further refined with the aim of maximizing the normalized mutual information. In order to investigate an improvement of the new optimization scheme, the authors tested the algorithm for both rigid and deformable cases using the established jaw bone (CT resolution: 4.57 *µ*m) [29] and cerebellum specimen datasets (CT resolution: 3.5 *µ*m, resized to 7 *µ*m in-silico) [21]. For the rigid jaw bone images, there was a clear improvement with a median error of 8.4 *µ*m. In the case of the deformable cerebellum specimens, the overall median distance between the landmarks resulted in 21.6 *µ*m. While this value is significantly higher, the authors argue that overall registration improved due to a limitation in the dispersion of the distances.

**Figure 6:**
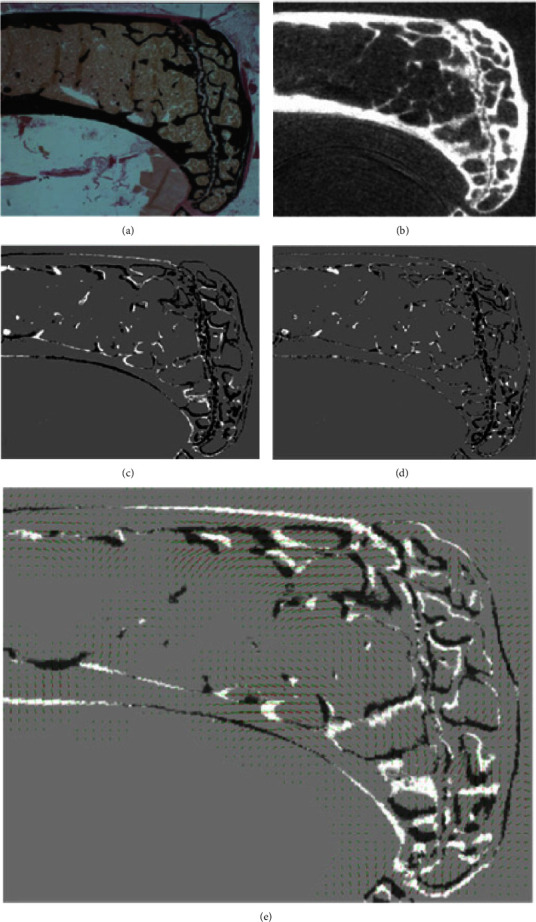
Comparison of the results achieved by SegReg and intensity-based registration for mouse tibia tissue [35]. Stained histological images (von Kossa staining) (a) and CT-plane (b) of mouse bone tibia were matched using SegReg. For this example, both affine and elastic registration techniques were applied. (c) The outcome of the affine registration approach. (d) The result of elastic registration. The overall difference was portrayed in (e) with the corresponding difference in the registration vector field superimposed. Reprinted by permission from Informa UK Ltd: Taylor and Francis online computer methods in biomechanics and biomedical engineering, 18 : 15, 1658–1673, histology to *μ*CT registration of 2D histological sections with 3D micro-CT datasets from small animal vertebrae and tibiae, Oleg Museyko, Robert Percy Marshall, Jing Lu, Andreas Hess, Georg Schett, Michael Amling, Willi A. Kalender, and Klaus Engelke, Copyright© 2014 Informa UK Limited.

Museyko et al. [35] matched micro-CT scans (15 *µ*m isotropic resolution) with histological images of vertebrae and tibiae obtained from different wild-type mice through segmentation-based registration (SegReg) and conventional intensity-based approaches and compared the results (Figure 6). For the SegReg, both modalities were first binarized and preliminary aligned based on visual inspection of corresponding landmarks. A secondary automated registration was implemented for a finer alignment. The intrinsic, i.e., intensity-based registration approach was not preceded by an additional segmentation step. The authors have chosen different complexities regarding the transformation of vertebrae (affine) and tibiae (elastic) specimens. Both registration methods were implemented using the insight segmentation and registration toolkit (ITK) [36].A B-Spline algorithm was used for a finer alignment. Mean Squared Difference (MSD) and Mattes Mutual Information (Mattes MI) [37] were applied as metrics for SegReg and intensity registration, respectively. Both approaches were compared using the Jaccard Distance, which is also known as the found exclusive disjunction (XOR-values). Quantification of the positional error of the slice in the volume was achieved by varying the position of the section by translating it into all three-dimensions and tilting it around the orthogonal axis based on six rotations. The resulting eight XOR-values were then computed, leading to a calculation of precision through root-mean-square (RMS) and standard deviation (SD). In this study, the accuracy of the resulting alignment could not be improved significantly for SegReg for affine registration approaches but showed that better results were obtained for elastic registration indicated by the decreasing overall registration error from 43% to 23%. Figure 6 compares the two registration methods investigated by [35] for the histological and micro-CT images of one mouse tibia.

The comparison of SegReg and the intensity-based approach was quantified using the standard deviation of eight specimens and the found exclusive disjunction, which was computed from the deviations in translation and rotation in eight positions. The authors found that the error in translation and rotation estimated by a standard deviation of the RMS value was lower in the SegReg (0.0039) compared to the intensity-based registration (0.1227) for translational errors in elastic registrations. The rotational error was noted to be an RMS of the standard deviation for both approaches of 0.0471 and 0.1189, respectively (micro-CT voxel size: 15 *µ*m; histology pixel size: 7.25 *µ*m). Furthermore, the authors computed and compared the offset for the affine and elastic registrations by comparing it to the best rigid transformation. This rigid transformation was created by neglecting the nonrigid components. The overall mean offset was computed to be 52 *µ*m.

A flexible framework for the non-rigid registration of individual histological two-dimensional images obtained from human brains to a three-dimensional MRI volume based on intensity-based criteria was proposed by Osechinskiy and Kruggel [38]. Their approach was designed specifically for cases in which there are only a sparse number of slides, but an MRI scan was performed beforehand. The framework was built up in a modular manner, starting with geometric transformation using a deformation model. First, a preliminary alignment of the slice in the bounds of the MRI volume was performed by translating and rotating the image by Procrustes or rigid transformation, resulting in nine and six degrees of freedom, respectively, with the assumption that no scaling was needed. Next, several options for the deformations can be chosen, namely, Thin Plate Splines (TPS) [39], Gaussian Elastic Body Spline Deformation Field [40], and B-Spline Free-Form Deformation Field [41, 42]. In a similar fashion, diverse options to calculate the similarity measure and optimization procedures were offered. The framework was assessed on MRI scans with an isotropic resolution of 0.35 mm × 0.35 mm × 0.7 mm and histological scans at a resolution of 12.7 micrometers/pixel. They demonstrated that the best performance was achieved by TPS in combination with a NEW Unconstrained Optimization Algorithm (NEWUOA) [43], optimization, and a correlation-coefficient-based cost function. The similarity between histological sections and MRI planes of human brains was measured by calculating the sum of seven coefficients [38].

### 2.2. Image-to-Image Registration (2D ⟶ 2D)

Image-to-image registration in the presented context refers to the alignment of the histology slice to the optimal plane inside the volume. Thus, concerns about geometrical integrity and optimal in-plane fitting of the three-dimensional bodies are discarded. However, after the accurate registration of both modalities, efforts may be put into the reconstruction of a virtual histological model.

#### 2.2.1. Nonelastic Approaches for Image-to-Image Registration (2D ⟶ 2D)

**Figure 7:**
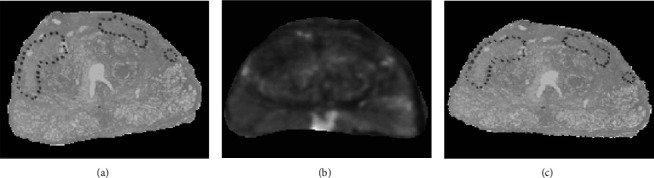
Aligned histological slide and MRI-plan of human prostate tissue through the group-wise scheme proposed by [10]. A histological slice of human prostate tissue (a) was through the group-wise alignment matched with a candidate MRI plane (b) and then affinely registered (c). The dotted polygons in (a) and (c) represent cancer cell distributions marked by experts. Reprinted from computerized medical imaging and graphics, 35/7–8, Gaoyu Xiao, B Nicolas Bloch, Jonathan Chappelow, Elizabeth M Genega, Neil M Rofsky, Robert E Lenkinski, John Tomaszewski, Michael D Feldman, Mark and Rosen, Anant Madabhushi, determining histology-MRI slice correspondences for defining MRI-based disease signatures of prostate cancer, Pages 568–578, Copyright (2011), with permission from Elsevier.

The optimal alignment of two images sourced by different modalities is a complex task, with a need to define a means of comparison. Furthermore, if two stacks of images are present, an individual correspondence needs to be established. While this correspondence may be defined by manual labor, it is a labor-intensive process, highly dependent on expert knowledge [10, 15, 21]. In order to overcome this problem and match histological slices and MRI slices of the human prostate, Xiao et al. [10] proposed the utilization of an iterative group-alignment scheme to estimate corresponding images and then individually compare the pixel value distribution of both modalities in opposition to the established pairwise comparison of two images. Their approach consists of three modules. First, all histological and MRI-images were separated into two groups according to the imaging modality (MRI or histology), with the goal of estimating a selection of MRI-slices that resulted in an optimal match based on the computed Mutual information (MI) [44, 45]. The *K*-top ranked matches were then averaged to distinguish between a probable and less likely match. While this step yielded an educated guess for the correspondence of both modalities, it did not account for distortion that might have occurred in the preprocessing of the tissue or the organ deformation experienced in the case of in vivo-MRI scanning with an endorectal coil. Therefore, the final alignment was conducted in an affine registration process, transforming the histological slice by calculating the MI of the two images and a simple optimization method. Xiao et al. [10] demonstrated that the proposed group-wise alignment results in a higher degree of similarity than pairwise comparison, e.g., a brute-force approach. This was evidenced by a lower proprietary error norm based on the L1-distance. According to their calculations, based on expert-generated ground truth, a pairwise alignment performs only half as well as their group alignment. Both approaches were assessed on the same data set with an MRI resolution of 0.27 mm/pixel. Their experiments showed that the group-wise alignment produced a smaller error than the pairwise comparison method.  Figure 7 shows a practical example of applying the group-wise alignment scheme to 5 *µ*m thick slices of human prostate tissue.

Quantified through their error norm, they measured values ranging from 0 to 2.7 for the group scheme and 1.7 to 5.2 for the pairwise scheme. While the pairwise matching set the individual slices in order, the complete generation of an authentic three-dimensional model still posed a challenge due to overlaps and offsets introduced to the slices during preparation. The authors addressed this problem with a 3D ⟶ 3D affine registration [10], which we present in a later section concerning higher dimensional registration.

#### 2.2.2. Elastic Approaches for Image-to-Image Registration (2D ⟶ 2D)

The correlation of histological slices with the scanned volume in an image-to-image registration approach relies on a priori knowledge of the section to plane correspondence, i.e., the position of the extracted histological image in the 3D-stack. If the order of sections is conserved during the preparation process, this a priori information may be used to establish such correspondence and may even allow for more sophisticated 3D ⟶ 3D registration approaches as presented by [46]. However, changes in orientation may need to be accounted for. The sectioning process can be further optimized through the inclusion of 3D-printed slicers that are created with an a priori scan of the tissue [13]. For example, Absinta et al. [47] showed qualitative improvements in the creation of serial sections of human brain tissue, allowing for the enhancement of the subsequent superimposition of 3D scan and histology. Turkbey et al. [48] segmented prostate tissue and generated 3D models with the use of the ANALYZE software (Mayo Clinics, AnalyzeDirect, Inc., Overland Park, KS, USA). Using the surfaces of these models, slicer molds were created and 3D-printed to allow sectioning without distortion.

Matching an individual plane to not only its adjacent neighbors but to a group of peers has been proposed [10, 49] in order to avoid the pitfalls of error propagation. One approach used by [49] involved the implementation of the feature point detection and feature description algorithm AKAZE [50]. Here, the feature points of the N preceding slices were matched symmetrically. Robust estimation of an affine transformation matrix was done by employing RANSAC [27]. In addition, the planned cutting plane was marked in the micro-CT volume beforehand, allowing for the allocation of corresponding images. Other approaches reconstruct the histological image stack through an initial alignment of each image to its en face or block face representation, referring to an image of the front side of a sectioned tissue (see, for example, [46, 51]). Manually placed landmarks in both histological and en face images of soft-tissue specimens of arteries with plaques were incorporated by Groen et al. [51] to register preand postprocessing recordings. The annotated landmarks were assigned through a B-Spline control point displacement [42] calculated by the means of MI and optimized using a gradient-descent algorithm. Next, the MRI and CT-slices were registered to the en face images to (i) determine the orientation of the slices and (ii) compensate for scaling errors that might have occurred. The former registration was performed as an automated rigid transformation, also incorporating MI as the similarity measure. Registration of CT- and en face images was based on manually edited landmarks using a rigid transformation as well as isotropic scaling. They reached an optimum of a 5-degree and 7-degree rotation error and a corresponding translational mean error of 0.6 mm and 1.2 mm (CT resolution: 18 *µ*m and histology resolution: 1.82 *µ*m).

In a two-step approach, Seise et al. [52] first affinely align pairs of binarized images of pig livers based on the relative overlap metric (Kappa-Statistic in ITK https://www.itk.org) [53]. The centers of the vessels depicted in the matched segmented images as well as interactively defined locations were then used as the initial group of corresponding points for TPS. Focusing on just the registration of histology to micro-CT, an average accuracy of about 0.5 mm (CT resolution: 0.4 mm) has been determined while the entire framework, including a 3D ⟶ 3D similarity transform between three-phase contrast-enhanced CT and micro-CT and 2D ⟶ 3D CT and histology registration, achieved a mean deviation of 2 mm. Considering the nature of the problem at hand, registering vessels, the presented approach produced satisfactory results when compared to its peers [54, 55], but limited applicability to other tasks can be assumed. Furthermore, the chosen implementation was a rather labor-intensive task.

Based on their previous work [1, 4], Albers et al. [5] used an inverted individual or overall color channel representation of the histological image to match the slide with the corresponding CT-plane. Following the aforementioned coarse matching using the Fourier-Mellin algorithm [56, 57], the fine alignment was achieved through the means of a B-spline deformation model implemented by elastic [9] and optimized through MI. The results were quantified using a displacement index [1] that computes the displacement and MI based on block matching. Ideally, this value should correspond to 0; however, for the case at hand, an overall value of 6.9 ± 2.0 was achieved for the transformed dataset, due to different image content shown in both histology and micro-CT. While the manual sectioning of the CT volume may complicate the reproducibility and applicability in an analog problem scenario, the overall result of the two-step approach produced a good outcome considering that the displacement was unlikely to equal 0 due to the different modalities involved.

Magee et al. [58] extended the intensity matching criteria approach by representing each pixel of each image with a feature vector constructed from the results of a Gaussian Filter on color and grayscale channels as well as texture features built upon their previous work [59]. This resulted in a common visual representation of images created through different modalities. These feature descriptors were labeled using prototypes and clustered together. Clusters were assigned to so-called tissue-classes that represent a multichannel representation of a given tissue using preexisting mapping functions. Next, a tissue class co-occurrence matrix of two mapping functions was generated. In order to quantize the similarity of said matrices, the MI was calculated and maximized by incorporating a greedy search algorithm. This process was repeated until each image was present in the same representation. Finally, the actual registration of the converted images was performed. Based on the idea of realizing a nonrigid registration by a set of rigid registrations on subimages, the images were first padded to the same size, rigidly aligned using phase correlation, and then divided into overlapping image pairs. These patches were superimposed by determining the rotation and translation offsets by calculating the phase correlation [60]. For each local registration, five transform vectors were created and overlapped with their peers using a least squares minimization method and subtracted from each other. The resulting vector set was then approximated by a B-Spline using a robust least squares-minimizing method [61]. These steps were repeated at different resolutions to achieve the best result. The authors employed their methodology to register MRI and histological images. This method outperformed iterative methods with an absolute error of 5.7 ± 5.8% in 100 *µ*m thick sections imaged at an MRI isotropic resolution of 50 *µ*m for specimens with low collagen quantification. Sections with a thickness above 100 *µ*m resulted in worse performance, with an error starting at 50% [59]. In their work, the others state that the achieved quality of registration was found to be within 200 *µ*m.

**Figure 8:**
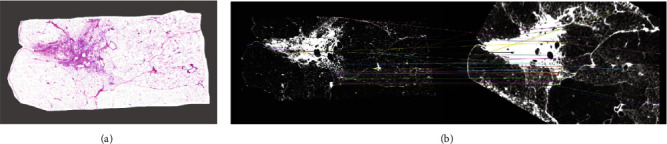
Matched histological slice with a CT-plane of a H and E-stained lung specimen following the method presented by Nagara et al. [49]. The original histological image (a) was converted into a grayscale representation and subsequently matched with the corresponding CT-plane based upon AKAZE features (b). The individual colors represent unique feature points identified in both modalities, which are connected by lines. Reprinted by permission from Springer Nature customer service center GmbH: Springer Nature patch-based techniques in medical imaging. Patch-MI 2017. Lecture notes in computer science vol 10530. Micro-CT guided 3D reconstruction of histological images, Kai Nagara, Holger R Roth, Shota Nakamura, Hirohisa Oda, Takayasu Moriya, Masahiro Oda, and Kensaku Mori., copyright© 2017 springer international publishing AG.

Approaches that already used feature points in a previous step logically tend to reuse them for a more advanced matching procedure. For completing the feature extraction of the CT-dataset, Nagara et al. [49] employed AKAZE as they have preceded to do for the histological images. Appointing the features of two corresponding slides as nodes of a Markov random field [62, 63], an elastic registration based upon normalized cross-correlation (NCC) was proposed. The resulting experiments showed promising results, with the best match quantified by a mean dice index of 0.744, a mean Jaccard index of 0.595, and a NCC of 0.608 (CT resolution: 49 *µ*m and 52 *µ*m; histological image resolution: 22 *µ*m).  Figure 8 illustrates the quantitative results of their approach by visually linking matched features.

Based upon the manually annotated landmarks, Katsamenis et al. [64] matched CT planes and histological slides according to the best visual correspondence in a preliminary step. In order to achieve a more accurate registration, an elastic vector-spline registration of the Fiji [65] Plugin UnwarpJ (available at https://bigwww.epfl.ch/thevenaz/UnwarpJ/) [66], implementation was used. The proposed method was realized in an almost exclusively manual workflow, which complicates its possible applicability to other problems. Furthermore, no indication of a similarity measure or accuracy quantification was provided.

### 2.3. Volume-to-Volume Registration (3D ⟶ 3D)

**Figure 9:**
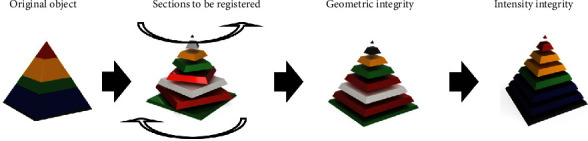
3D-volume reconstruction based on subsequent image-to-image registration poses a challenge in the conservation of the geometric consistency of the sectioning tissue. In order to register a histological image with a scanned volume, the individual sections first need to be aligned together, e.g., the volume needs to be reconstructed while geometrical consistency is maintained. Consecutive registration of individual slices needs to be performed until the original shape of the sectioned tissue is reconstructed in silico. Subsequently, inconsistencies in the intensity can be accounted for.

A full registration of two three-dimensional models poses the most challenging approach for multimodal image fusion and is commonly composed of a multistep workflow in which lower-level matching methods are applied iteratively. Consistency with regard to the geometrical integrity or geometry consistency of the resulting model is a frequently addressed problem that is visually noticeable in layer shifts found in the *z*-axis, in cases of curved objects, also referred to as the “banana effect” [67, 68]. This could result from a sparse number of histological slices as well as distortion or lesions introduced during the slicing process. Furthermore, changes in the intensity of the aligned planes or sections need to be accounted for. A visualization of the problem at hand is presented in Figure 9.

The following research tackles the problems of (i) reconstructing the specimen from the slices and (ii) matching this 3D representation with a scanned model. It is worth mentioning that the topic of 3D virtual histology has been addressed by other authors who did not rely on a scan of the specimen as a ground truth and therefore did not implement multimodal registration as evidenced by [69–71].

Full 3D ⟶ 3D registration could contribute and expand on existing approaches through the almost exact correlation of 3D scan and histology. With the addition of a registration of the reconstructed histology volume and an MRI scan in the workflow of Turkbey et al. [72], future research may produce an increased degree of correlation between both modalities, thus providing further aid in the diagnostic process.

#### 2.3.1. Nonelastic Approaches for Volume-to-Volume Registration (3D ⟶ 3D)

**Figure 10:**
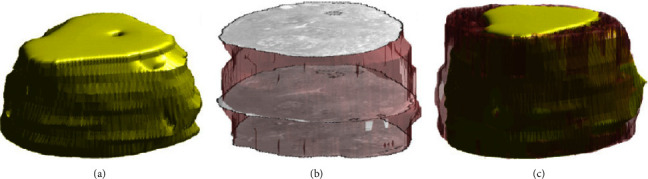
Expanding on their group-wise image-to-image registration, Xiao et al. [10] undertook efforts to reconstruct the original tissue in silico. The rendered MRI volume (a) was registered with the reconstructed histology volume (b) of the human prostate. The resulting joint volume is portrayed in (c). Reprinted from computerized medical imaging and graphics, 35/7–8, Gaoyu Xiao, B Nicolas Bloch, Jonathan Chappelow, Elizabeth M Genega, Neil M Rofsky, Robert E Lenkinski, John Tomaszewski, Michael D Feldman, Mark Rosen, Anant Madabhushi, Determining histology-MRI slice correspondences for defining MRI-based disease signatures of prostate cancer, pages 568–578, copyright (2011), with permission from Elsevier.

In addition to the established group-wise alignment scheme explained in the previous section, Xiao et al. [10] used the plane and slice correspondence to create a three-dimensional representation of the histological image stack and registered it to the volume. Retaining the interslice distance of the MRI-scan as well as the order of the individual slices, they created a pseudo-volume of the stained tissue. Missing values were compensated for by zero-padding, essentially leaving absent parts blank. In order to align both volumes, the MRI was used as the transformed volume in an affine registration process. The authors opted to avoid overfitting and therefore deliberately chose a nonelastic approach. As they have done for their 2D ⟶ 2D registration algorithm, MI and a dedicated simplex optimization approach were applied. The authors did not state the achieved margin of accuracy or similarity for the implemented 3D registration. A qualitative example of their results is portrayed in Figure 10.

#### 2.3.2. Elastic Approaches for Volume-to-Volume Registration (3D ⟶ 3D)

After the histological processing of the tissue specimen and subsequent imaging, a three-dimensional virtual model reconstructed from the individual slices needs to be obtained in order to match it with the volume. This can be achieved by registering the histological images to their respective block face or en face representation based upon an MI implementation [37, 44, 45, 73] as a basis for 3D Registration and the 2D ⟶ 2D approaches described in the image-to-image registration section of this review. In the case of Mancini et al. [74], whole brain specimens were divided after an MRI scan to realize a histological section of smaller blocks. To preserve the overall anatomical structure of the brain, these blocks were matched to whole-slice photographs using SURF [24] and RANSAC [27]. After obtaining the full virtual histological model, a 3D registration was proposed by first resampling the MRI of the brain according to the block orientation. Then, each slice pair was coarsely matched by the means of NiftyReg [75], realizing a nonelastic transformation. A subsequent fine alignment was implemented through stationary velocity fields [76].

**Figure 11:**
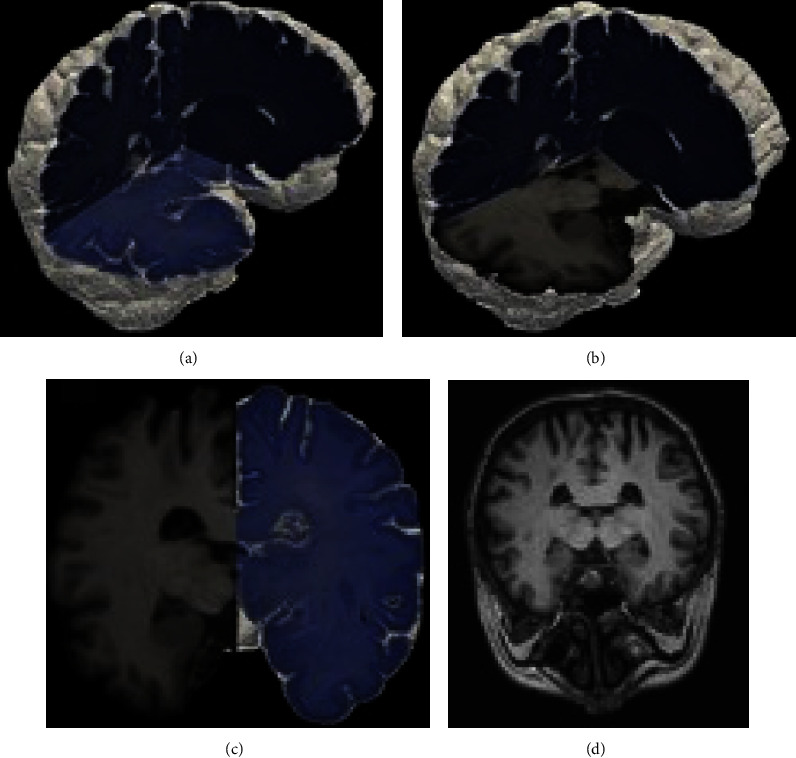
Comparison of the reconstructed three-dimensional histology with the MRI volume of a whole postmortem human brain [46]. The complete reconstruction of the histology volume (a). (b, c) The correlation of both modalities. A reference of the MRI (d). Reprinted from IEEE conference on computer vision and pattern recognition workshops (CVPRW), 2016, Maryana Alegro, Edson Amaro, Burlen Loring, Helmut Heinsen, Eduardo Alho, Lilla Zöllei, Daniela Ushizima, Lea T Grinberg, multimodal whole brain registration: MRI and high resolution histology, pp. 634–642, copyright (2016), with permission from IEEE.

Alegro et al. [46] realized their 3D registration through asymmetric diffeomorphic registration [77]. Facing the ill-posed problem of geometrical altering of the tissue during processing, they propose to preserve the geometrical integrity of the virtual histological image stack by 2D registering the sections to an a priori ground truth captured in block face images of the whole postmortem human brain acquired during sectioning. The authors claimed that their methodology prevents shifts in the geometrical integrity, i.e., the *z*-effect. The registration was implemented using advanced normalization tools (ANTs) [78] and an affine registration algorithm based on Mattes MI [37]. Following intensity correction and resampling, both 3D-stacks were then registered. Diffeomorphic nonlinear registration [77, 79] was implemented to compensate for artifacts present in the histological samples with Mattes MI [37] as the matching criterion. Their results were quantified using the Dice-coefficient [12] and yielded experimental results of 0.59, 0.65, and 0.75, respectively, with a relatively low MRI isotropic voxel resolution of 1 mm^3^. A visual representation of the reconstruction of a three-dimensional histological model of a brain specimen is displayed in Figure 11.

Rusu et al. [80] fused histological images and MRI scans of lung specimens to detect features of pulmonary inflammation based on three-dimensional registration. In a scheme with increasing complexity, the authors first rigidly aligned neighboring histological slices based on MI. Using elastix [9], a virtual histology model was reconstructed through registration with an ex-vivo scan of the lung, thus limiting the spatial deformations introduced during sectioning. Utilizing a three-level pyramid affine registration optimized by MI as the scoring function, the processes were realized. Next, the histological volume was registered to an in vivo scan by affine and deformable transformation. The alignment was further optimized with regard to the individual lobular units extracted from the specimen. Utilizing the entire gamut of the information held by the histological images, both volumes were further merged, allowing for the mapping of pulmonary inflammation onto the in vivo scan. The histological model was warped onto the in vivo-MRI scan using a B-spline based elastic registration based on a three-level registration scheme to optimize MI. With an ultimate grid spacing of 4 mm, a final alignment error of 0.85 ± 0.44 mm (root-mean-square deviation between the 17 landmarks) (in vivo-MRI resolution: 250 *µ*m, histological resolution: 0.75 *µ*m) was achieved.

In a vastly different approach in comparison to the prior research concerning matching criteria and implemented metrics, Lee et al. [81] reconstructed the surface of a cochlea based on histological images and CT scans by using an iterative closest point (ICP) algorithm [82]. Both modalities were processed in order to generate a wireframe representation of the surface. Substructures of these wireframes, constructed of triangular faces, were used to identify corresponding markers and subsequently match them using the ICP algorithm. In an iterative process, the optimal deformation function to map these points onto another was determined by minimizing the distance between the sum of all points. The process was optimized by observing the RMS value between successive slides. After partial alignment, the entire surface was matched connecting the individual wireframes. This final registration was done using an affine transformation. The mean distance between two points in the reconstructed surface model was calculated for several RMS and averages to about 0.0805 mm (micro-CT resolution: 30 *µ*m; histology images: 300 pixels/in (about 12 pixels/mm)).

## 3. Comparison of the Presented Registration Approaches

This summary presents a prevalent excerpt of registration strategies to implement an optimal alignment transformation of a 2D histological image with the analog three-dimensional plane representation. Given that the analogous objective is the multimodal registration of complementary imaging techniques, we believe that a broader scope can aid the implementation of an akin solution. For the purposes at hand, accuracy in terms of the quality of alignment is paramount. Due to the authors' different mission statements manifested in the dimensionality of the registration method, a variety of targeted types of tissue, and the desired outcome, a variety of metrics were chosen to estimate the individual performance. With different equipment and software used by the authors for imaging and preprocessing, a universal solution through direct inspection and subsequent appointment is ambiguous. However, with a quantitative comparison of the selected research, a trend for a subset of algorithms can be observed. Through the found variety of similarity measures and stated accuracy, we quantified the performance of the individual approaches, for the cases where it was possible, by weighing them with the stated resolution of the three-dimensional imaging technique and calling this relative accuracy. Since histology imaging has a higher native resolution, the a priori MRI or CT scan can be characterized as the limiting factor. In Tables 1–3, the presented research, according to the established dimensionality categories, is summarized by their achieved similarity, resolution of the scan, and relative accuracy. This accuracy is computed with the 3D isotropic resolution and the found similarity measure, therefore, allowing us to directly compare the precision of the given approach concerning the multimodal registration of histology and scan if measurements with equal units are provided. Possible results range from 0 to 1, with 1 being the optimal relative accuracy. Through this comparison, Tables 1–3 were compiled to create an overview of the achieved performance. Starting with Image-to-Volume registration approaches, the presented literature is quantitatively compared by the reported performances of the described approaches and put into context with the limitations of the three-dimensional scan. We documented this effort through the introduction of a relative accuracy that is computed as the ratio of the found similarity (depending on the values stated by the authors) and 3D isotropic resolution in Table 1.

Due to the different approaches the authors took to quantify their results, a clear comparison through the computed relative accuracy is not always feasible. However, among the three papers in which sufficient measurements were provided, a distinct difference in the performances is observed. Since these papers provide their found accuracy as plain distances measured in *µ*m, a calculation of the relative accuracy is possible. In Table 2, we applied and listed the found similarity and isotropic resolutions and, if possible, calculated the relative accuracy, which we have been using as a means for quantitative comparison but now apply to the image-to-image registration cases presented in this paper.

For the problem of 2D ⟶ 2D registration, a comprehensive comparison is rather difficult to establish due to the coarse isotropic resolution of the scans. This manifests itself as a second factor to be considered when choosing a registration approach. One should be aware of the stated resolution when choosing an approach for proprietary problems. Finally, in Table 3, we list the observed performance of the volume-to-volume registration approaches taken from the individual publications, respectively.

A comparison of the provided data was not possible for the 3D ⟶ 3D. Either the data was not provided or not applicable for the calculation of the relative accuracy. Furthermore, there is less research being conducted in this domain possibly due to the complexity of the task.

## 4. Conclusion

The herein presented review of image registration algorithms aims at providing a broad overview of techniques that can be used for the registration of histological slides with 3D-imaging modalities such as CT and MRI. Since there is a strong interest and intensive research in this field, we focused on reports published in the last ten years. The publications were sorted based on the complexity of the transformation, allowed degrees of freedom and the dimensionality of the problem at hand. Here, a clear discrepancy in the amount of research published was observed, with 2D ⟶ 2D and 2D ⟶ 3D being significantly more prominent than 3D ⟶ 3D applications. The latter field seems less well understood given the limited information about the used algorithms and the focus on very specialized use cases. Furthermore, we conclude that even though microscopic images of processed hard-tissue typically show fewer deformations than observed in soft-tissue histology, dedicated algorithms for this specific task are less prominent in the literature. Instead, a majority of elastic solutions are presented for precise superposition, while, on the other hand, nonelastic methods are primarily used for preliminary alignment of both modalities. A majority of the presented publications that deal with complex slice-to-volume or volume-to-volume registration strategies divide the process into distinct substages: (i) three-dimensional registration is initiated by a priori matching of the corresponding planes, resulting in transferring the original 3D problem into a 2D ⟶ 2D registration problem, (ii) typically iterative refinement of the position of the histological section in the scanned volume is applied to increase the precision, and (iii) finally, for 3D ⟶ 3D registration, an additional arrangement step is utilized to match both structural and geometrical properties.

In order to loosely compare the performance of the proposed strategies, we calculated the relative accuracy based on the stated matching error in relation to the lowest spatial resolution of the used image datasets. This proved to be practical for most nonelastic approaches; methods employing more sophisticated similarity measures or novel quantification strategies can hardly be compared in this way due to their heterogeneous nature and the involvement of complex alignment schemes. Also, in the case of elastic registration, our simple comparison metric cannot be applied. Nevertheless, we found large variations in the achievable relative accuracy and hope that this information will help the reader pick the ideal technique for his/her application.

Taking the above-declared limitation into consideration, we, however, observed that for the literature considered in this review, a clear tendency to favor the use of intensity-based approaches generally tends to perform better than their feature or landmark-based counterparts. However, this might change if feature-based image processing methods are incorporated with registration approaches, which are predominantly realized by intensity matching. The visible deformation introduced to the specimen during histological sectioning will continue to be a major hindrance for extraction algorithms.

Overall, we observed the dominance of relative accuracies or measures instead of a transparent distance quantification (e.g., in micrometers). However, a set of standardized methods to quantify the resulting alignment of two images after the registration may hold the key to efficiently establishing a unique approach that could be suitable as a commonly recognized means to evaluate the quality of a registration approach and thus allow for direct comparison of the individual algorithm's performances.

- *Vectorized Norm.* Prior knowledge of individual landmarks and points of interest can be obtained, or expert knowledge is provided. A vectorized norm may be provided to (i) determine the overall performance in slice correspondence [10] or to (ii) normalize the deviation between two markers present in both images [11]. While plain distance measurements are also feasible, the norm approach should also be considered if the registration methodology is based on feature descriptors or if the image is transformed.
- *Set Theory Approaches*. If no prior knowledge of plane correspondence is available, methods based on logic operations may be used. Quantification of alignment for intensity values through Intersection of Union, i.e., Jaccard index [34], provides a performance statement based on the resulting overlap which can be expressed in percentages or in values ranging from 0 to 1. Therefore, a universal comparison could be achieved by estimating the similarity and difference in pixel-based intensity values after the two images are superimposed.
- *Benchmarking*. In image processing applications and machine learning, benchmark-data sets have been the gold standard to verify and validate the performance of dedicated algorithms, e.g., [83–86]. Typically, these images stem from real objects or were artificially generated. If such a multimodal dataset, with constant resolutions and unspecific staining protocols, including expert-based ground truth and a defined set of metrics, is to be established, future work needs to be conducted based on the achieved performance. Using such benchmark data could be used to verify and validate results found by other researchers who already considered expert-based matching as ground truth, e.g., [21] in an attempt to reduce inductive bias. A ground truth-based evaluation set for the benchmarking for the reconstruction of 3D-volumes by 2D ⟶ 2D registration has already been proposed by Lobachev et al. [87]. Furthermore, different staining protocols need to be accounted for. This problem is currently being tackled by the participants of the Automatic Nonrigid Histological Image Registration (ANHIR) Challenge [85, 86, 88, 89].

This review shows that the registration of hard- and soft-tissue histology to a prior generated 3D scan of the specimen is of broad interest. However, each of the presented approaches differs not only in the pursued goal but also in the registration method. Thus, a comprehensive comparison of performance and accuracy can only be achieved with great difficulty. This underlines the need for a general quantification method and an agnostic procedure to compare and evaluate each workflow objectively. With this review, we hope to provide researchers new to the field of image registration an easy decision tree to pick the optimal strategy for their registration problem. In analogy to the structure of this review, one should first be aware of the dimensionality of the problem to be tackled and then decide how severe the alterations introduced to the tissues are and finally decide on the metric which promises the best optimization opportunity.

## Figures and Tables

**Table 1:** Quantitative comparison of different slice-to-volume registration approaches through the calculation of the relative accuracy.

Author	Matching criteria	Stated similarity	3D resolution (per pixel)	Relative accuracy
Lundin et al. [14]	Feature	106.3 *μ*m	10.7 *μ*m (nominal)	0.1
Becker et al. [11]	Landmark	*L* score: 91/100	8.6 *μ*m (nominal)	—
Chicherova et al. [20]	Feature	250 *μ*m	4 *µ*m	0.02
Chicherova et al. [22]	Intensity	21.6 *µ*m	7 *µ*m	0.3
Khimchenko et al. [21]	Feature	4 *μ*m	0.9 *μ*m	0.2
Museyko et al. [35]	Intensity	XOR-score	15 *μ*m	—
Osechinskiy and Kruggel [38]	Intensity	Similarity measure of CC, LC, RC…	350 *μ*m × 350 *µ*m × 700 *µ*m	—

**Table 2:** Quantitative comparison of the stated similarities and resolution for the image-to-image strategies taken from the literature discussed.

Author	Matching criteria	Stated similarity	3D resolution (per pixel)	Relative accuracy
Xiao et al. [10]	Intensity	L1-norm	270 *µ*m	—
Nagara et al. [49]	Feature	Dice index, Jaccard index, recall, and NCC	Dataset 1 : 49 *µ*m, dataset 2 : 52 *µ*m	—
Groen et al. [51]	Intensity	600 *µ*m (best value used)	18 *µ*m	0.03
Katsamenis et al. [64]	Landmark	—	8.84 *µ*m	—
Seise et al. [52]	Intensity/landmark	500 *µ*m (worst value used)	400 *µ*m	0.8
Albers et al. [5]	Intensity	Displacement index	2.33 *µ*m	—
Magee et al. [58]	Intensity	200 *µ*m	50 *µ*m	0.25

**Table 3:** List of volume-to-volume registration strategies discussed in this review. Due to a lack of information, a direct comparison according to the relative accuracy was not possible since only Rusu et al. [80] and Lee et al. [81] provided sufficient data. Therefore, there can be no quantitative evaluation.

Author	Matching criteria	Stated similarity	3D resolution (per pixel)	Relative accuracy
Xiao et al. [10]	Intensity	L1-norm	0.27 mm	—
Rusu et al. [80]	Intensity	0.85 mm	250 *µ*m	0.3
Mancini et al. [74]	Feature	—	200 *µ*m × 200 *µ*m × 400 *µ*m	—
Alegro et al. [46]	Intensity	Dice 0.59, 0.65, and 0.75	1 mm	—
Lee et al. [81]	Landmark	850 *µ*m	30 *µ*m (maximum resolution)	0.04
